# Sense of Presence and Subjective Well-Being in Online Pet Watching: The Moderation Role of Loneliness and Perceived Stress

**DOI:** 10.3390/ijerph17239093

**Published:** 2020-12-05

**Authors:** Zekun Zhou, Duo Yin, Quan Gao

**Affiliations:** 1 College of Tourism and Service Management, Nankai University, Tianjin 300350, China; zhouzekun.0327@163.com; 2 Higher Education Mega Centre, School of Geography and Remote Sensing, Guangzhou University, Guangzhou 510006, China; 3 School of Social Science, Singapore Management University, 90 Stamford Road, Singapore 188065, Singapore

**Keywords:** telepresence, social presence, flow experience, subjective well-being, loneliness, perceived stress, pet videos/livestreams

## Abstract

Watching videos/livestreams concerning pets is becoming an increasingly popular phenomenon among youth in China, thus the social dynamics and psychological impacts of this pet-centred online activities worth in-depth exploration. This study investigates the sensual experiences of the audiences who have watched pet videos/livestreams and examines how these online experiences influence their subjective well-being. We develop a conceptual model that bases on the relationships between telepresence, social presence, flow experience, and subjective well-being to comprehend this mechanism. The result of 439 samples indicates that both telepresence and social presence have significant positive effects on flow experience, and social presence also has a positive impact on subjective well-being. We also examine the role of loneliness and perceived stress in moderating the effects of online pet watching on subjective well-being, showing that loneliness has a significant positive moderating effect on the relationship between social presence and flow experience, while perceived pressure has a negative moderating effect on the relationship between telepresence and flow experience. This study not only demonstrates the positive effect of an online pet on subjective well-being and but also uses interview data to comprehend the social processes underlying this effect. We also discuss the theoretical and practical values of this study in improving public health in the digital age.

## 1. Introduction

In recent years, the rapid development of information technology and the proliferation of mobile devices have profoundly affected the ways that our everyday life is organised and made sense of, especially in terms of the leisure activities [1] and public health [2]. It is important to note that we are all living in a digital age [3]. According to the statistics of China’s Ministry of Industry and Information Technology (MIIT), China has the world’s largest population of Internet users (1.32 billion) and mobile phone subscribers (1597 billion) as of July 2020. An increasing number of people have engaged in digital practices and space for leisure (e.g., YouTube, TikTok). As an extension of offline leisure activity [4], digital leisure’s impacts on subjective well-being have been found [5] and recognised [6]; however, the more nuanced dynamics regarding how digital leisure affects individual subjective well-being is still under-explored. Existing research has noted that subjective experience is the centre of media enjoyment. In particular, the notions of “flow” the state of mind characterized by concentration and enjoyment stimulated by sensory materials [7]) and “presence”(the sense of ‘being there’ in a mediated environment [8]) are widely used to describe the sensual subjective experiences elicited by online activities [9]. In particular, the sense of presence is an important aspect of the engagement experience [10], as well as an important variable in predicting online enjoyment [11]. Nevertheless, how different forms of digital experiences mutually shape one another and their various effects on subjective well-being are still unclear [12].

In modern society, pet animals have played an increasingly important role in human society and pet owners regard them as children and relatives that they spend time, energy, and money on [13,14]. A white paper on Chinas pet industry in 2019 showed that the number of pet dogs in Chinas cities and towns was 55.03 million, and the number of pet cats was 44.12 million by 2019. The market generated by pets and pet owners reached 202.4 billion yuan. It is acknowledged that pet ownership can bring health benefits and provide a source of companionship [15]. Previous studies have found that interacting with pets has positive effects on people’s well-being, including helping people maintain regular exercise and a healthy diet [16], improving blood pressure [17], and reducing the risk of cardiovascular disease [18]. And pet ownership also had a positive effect on mental well-being [19], pet owners showed significantly lower perceived stress [18,20,21,22], less anxiety [23], and higher self-life satisfaction [24]. In addition, contact and interactions between pet owners can provide more social connections, social cognition, and social supports to owners [25,26], especially the urban empty nesters [27]. However, contemporary urban people generally live in situations characterized by continuously moving around, strong pressures, and busyness. They do not have enough time and energy to take care of pets. Moreover, with lower wages and smaller living space, the youth cannot afford keeping pets by themselves, although they still want to get close to, and interact with, pets. Therefore, in response to these desires, pet online streaming has emerged in the past five years in China, particularly among young people. According to a recent report released by Taobao, which is one of the largest online retail platforms in the Asia-Pacific region, one million people watched pets live streaming on Taobao every day, and the number of pets live streaming on Taobao increased by 375% year-on-year in February. Accordingly, “online pet addicts” becomes a buzzword in China’s Internet to describe the audiences who engage online pet streaming. However, the social dynamics of this social phenomenon, and in particular, its influences on people’s well-being has not yet examined. 

Research on the impact of pets on people has been abundant, and pets’ positive effects on human health have been widely recognized by scholars. However, whether the online pet can also have a positive psychological impact remains unknown. This study focuses on online pet streaming, an emerging digital leisure mode, and tries to explore whether online pets have a positive impact on people’s subjective well-being and how it works. Based on the theory of presence, this study develops a conceptual model based on telepresence, social presence, flow experience, and subjective well-being. We then examine it by using structural equation modelling and hierarchical regression with actual survey data. We also use the interview data from pet online streaming watchers to interpret the operation results so as to uncover the social processes related to online pet watching. This study suggests that both telepresence and social presence have positive effects on subjective well-being, and flow experience plays an important role among them. Moreover, while it is noted that the people in modern societies have found to be more lonely [28] and more stressed [29], we also examine if loneliness and perceived stress influence the extent that people engage watching pet videos/livestreams as a way to cope with their feelings. This paper tries to enrich the digital leisure studies and extend the research on the impact of pets on people into the digital space of the Internet.

## 2. Literature Review and Hypotheses

**Figure 1:**
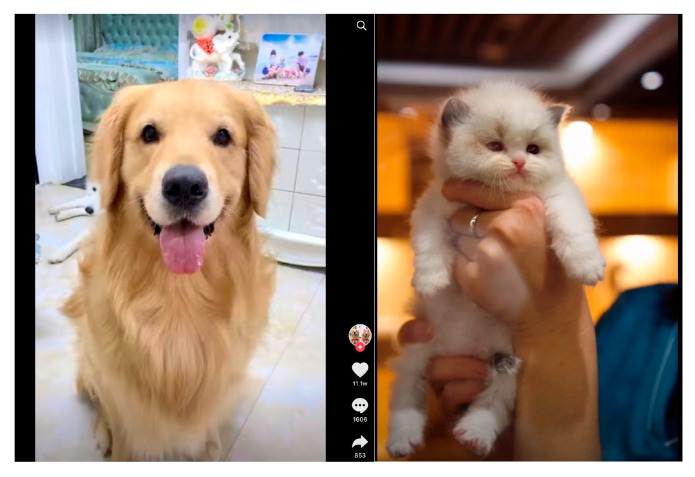
Examples of pet video and pet live streaming.

As showed by social medias, pet online streaming has become a widely participated activity among young people (see Figure 1). Watching entertainment videos can trigger strong positive emotions [30], and online pet videos/livestreams is one type of entertainment videos. Myrick [31] found that browsing online cat media may improve emotional well-being. And such media is highly accessible, and that is why an increasing number of youth participate in online pet watching and gain pleasure and happiness from it.

### 2.1. Presence and Flow Experience

In a digital society, social media largely influences the way that people make sense of the world. Thus, researchers began to explore the illusion that exists in computer-mediated remote spaces and proposed the concept of presence to describe the sensual feelings and experiences of audience who had engaged in digital remote spaces (such as chatrooms, online games, etc.). The concept of presence is derived from the field of virtual experience, that is, the sense of presence in a remote environment. Steuer believes that presence is “a mediated perception of the environment” [32]. Current mainstream academic research argues that presence sensing mainly includes two types: telepresence and social presence [8]. “Telepresence” is also known as virtual presence [33], and it was originally proposed by researchers in the fields of engineering and computer science. It is the general term for an individual’s own perception in an artificial or remote environment, which describes a subjective experience of an individual in a virtual environment [34], and specifically refers to the ability of an individual to imagine a geographic location in a virtual space. Social presence is an individual’s perception of the results of interpersonal communication. It describes an individual’s awareness of being with others and specifically refers to the sense of being with others. In other words, telepresence reflects a user’s feeling of “being there”, and social presence reflects the feeling of “being with others”. In the past ten years, academic research on presence has continued to deepen, mainly in the areas of online learning and marketing. 

Existing studies show that the use of social media deeply affects people’s perception of the quality of life and happiness. Well-being has been extensively associated with social bonds and contacts [35], and lacking social connection is a major risk factor for unhappiness [36]. As an important indicator to measure media engagement, presence has an important influence on the experience of digital leisure. Therefore we propose the following hypotheses:

**Hypothesis** **1** **(H1).** *While watching pet videos/livestreams, telepresence has a significant positive effect on the subjective well-being*.

**Hypothesis** **2** **(H2).** *While watching pet videos/livestreams, social presence has a significant positive effect on the subjective well-being*.

The concept of flow originated from the flow motive theory proposed by Csikszentmihalyi [37]. Flow experience refers to the emotional state of pleasure and excitement generated by mental stimulation when people concentrate on their own activities [38]. The flow experience has two attributes—concentration and enjoyment [39]—and people in the flow experience may be wholly absorbed, losing sight of surroundings and losing touch with time, space and even the self. Scholars have studied the flow experience in a variety of Internet activities and showed that the flow experience significantly affects the perception, attitude, behavioural intention or behaviour of Internet users, and it may result in social media enjoyment and inner pleasure [40]. Researchers have found connections between presence and flow in a virtual environment [41], and telepresence can be seen as a contributing factor to flow [42,43]. Both presence and flow look similar; however, there is a clear boundary between them. As Brockmyer et al. [12] said, flow may be a deeper state of engagement with media than presence because it involves experiencing an altered state. In other words, the more that participants feel immersed in the simulation experience, the greater the likelihood that they will enter a state of flow, as the saying goes: “I feel present, therefore, I experience flow” [44].

In addition, according to media richness theory, live streaming and short videos can be regarded as the social media with highly rich sensual contents. Video information has the characteristics of a large amount of information, vivid, intuitive and specific, and live video also has real-time and interactive attributes. Therefore, live videos can make people feel more immersive, more authentic, and more interactive, which means people could feel a high level of presence when they are watching pet videos and more likely to have a flow experience. Therefore, this research proposes the following hypotheses:

**Hypothesis** **3** **(H3).** *While watching pet videos/livestreams, telepresence has a significant positive effect on the flow experience*.

**Hypothesis** **4** **(H4).** *While watching pet videos/livestreams, social presence has a significant positive effect on the flow experience*.

### 2.2. Subjective Well-Being

With the development of positive psychology, researchers have recently paid increasing attention to subjective well-being, and subjective well-being has become an important psychological indicator to measure quality of life. Diener defines subjective well-being as people’s cognitive evaluation of their own life satisfaction [45], and measures of the quality of life or life satisfaction are used to assess subjective well-being. With the current development of information and communication technology (ICT), people’s subjective well-being and life satisfaction are increasingly closely related to the use of mobile devices and digital leisure enjoyment.

Leisure participation has obvious benefits on emotional well-being [46], can promote a positive mental state, and can promote the accumulation of psychological resources [47]. Research has shown that leisure participation is beneficial to an individual’s emotional health in daily life, and it has the ability to buffer negative experiences in everyday life [48]. According to flow theory, scholars regard leisure activities as specific activities that produce flow experience. People engage in leisure activities for the purpose of relieving stress and achieving flow experience [49]. Flow experience can promote positive emotions and happiness. In other words, the higher the flow experience is, the easier it is for people to feel happiness [50]. Rogatko notes that flow experience is an important factor in obtaining happiness [51] and flow has been found to have a positive relationship with subjective well-being [52,53]. Therefore, we propose the following hypothesis:

**Hypothesis** **5** **(H5).** *While watching pet videos/livestreams, flow experiences have significant positive effects on subjective well-being*.

### 2.3. Loneliness and Perceived Stress

The term “loneliness” originally came from the field of medicine and was introduced into social psychology by Weiss in 1973. He believes that the concept of loneliness is a negative experience caused by people’s unsatisfied needs [54], which is based on “demand theory”. The second representative concept of loneliness was proposed by Peplaue and Penman, which was based on “cognitive theory”. This definition implies that when people subjectively realize that there is a gap between the expected value of their interpersonal relationships and the actual achieved value, the experience of loss and unpleasantness is triggered [55]. The Internet is a virtual behavioural environment, but it is real people who interact in this environment. The interactive behaviour of using bullet screens and sending messages can both be seen as “quasi-social interactions”.

Many studies have shown that company of animals has positive effects on people’s physical and mental health. Interactions with companion animals can assist people in coping with loneliness and depression, and pet ownership and pet attachment can help people reduce feelings of loneliness [56]. However, whether the level of loneliness affects people’s emotions about pets has been less examined. People with high loneliness traits prefer social network interactions over people with low loneliness traits and use this behaviour to compensate for the lack of offline interpersonal communication. Therefore, the author considered that people under different loneliness conditions may show different levels of presence and flow experience when they watch the animal videos and livestreams. Therefore, this research proposes the following hypothesis:

**Hypothesis** **6** **(H6).** *While watching pet videos/livestreams, loneliness will moderate the relationship between social presence and flow experience*.

Perceived stress is a process in which individuals identify and assess whether perceived stimuli threaten the individual or produce meaning. Therefore, the sense of stress is the individual’s psychological response after identifying and evaluating threat stimuli in the environment. From the physiological point of view, the sense of stress refers to what an individual faces regarding external environmental stimuli associated with tension and discomfort, which will lead to an individual feeling threatened and even out of control. On the ground of the theory of attentional resource limitation proposed by Kahneman [57], attention is a kind of limited cognitive resource. The processing of stimuli requires cognitive resources. The more complex the stimulus or the more complex the processing, the more cognitive resources are occupied. Broadbent [58] believes that when a person pays attention to more than one stimulus, they are limited by attention capacity. This limitation comes from an internal filter, which makes attention accept some stimulus information and reject other stimulus information. Therefore, in conditions of greater pressure, more psychological resources are used to deal with psychological emotions, while the ability to process external information will be reduced, which will affect people’s sense of presence and involvement while watching pet videos or livestreams. We propose the following hypothesis:

**Hypothesis** **7** **(H7).** *While watching pet videos/livestreams, perceived stress will moderate the relationship between telepresence and flow experience*.

**Figure 2:**
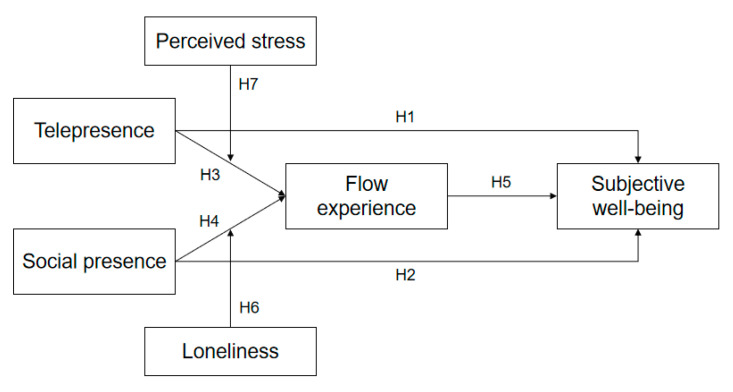
Model of the proposed hypotheses.

These hypotheses are summarized in Figure 2.

## 3. Materials and Methods

### 3.1. Measures

The questionnaire consists of two parts. The first part includes the demographic characteristics and behavioural characteristics of the participants, including gender, age, monthly income, education level, watch time, viewing duration, etc. The second part consists of the measurement model scale, including telepresence, social presence, flow experience, subjective well-being, loneliness and perceived stress. All of the measurement items were adapted from previous studies, with slight re-wording to capture the uniqueness of the video entertainment context. First, telepresence was measured using the scale developed by Kim and Biocca [59]. This scale was developed in virtual environments and is truly useful for media theory. Second, the social presence of watching videos was operationalized as the extent to which consumers perceived a sense of human warmth, sociability and excitement from pet videos, and we used Gefen’s scale [60] to measure social presence. Third, flow experience was measured using the scale developed by Ghani and Deshpande [39]. “Flow” is considered to be an important notion for understanding the experience of netizens and is characterized by a high degree of concentration and enjoyment. Fourth, for subjective well-being, we drew from the life satisfaction measurement method of Sirgy et al. [61], using 5 questions to measure. Fifth, loneliness was measured by the scale developed by Hughes, in the paper “A short scale for measuring loneliness in large surveys” [62], using three items that appear to measure overall loneliness quite well. In addition, we used 1 item to measure perceived stress: “In the last month, how often have you felt that you were unable to control important things in life?”, which was from Cohen’s perceived stress scales [63]. The rating of each item was based on the Likert 5-point scale, ranging from 1 (strongly disagree) to 5 (strongly agree).

### 3.2. Participants and Procedure

The data in this paper were obtained from a questionnaire sampling survey. Because the purpose of this study was to investigate people’s actual feelings and experiences in watching pet videos on mobile terminals, using a self-report questionnaire is appropriate. An online questionnaire was used to ask participants who have watched pet videos about their real thoughts on related issues based on their actual experience. The authors used wenjuanxing (https://www.sojump.com), which is a professional online survey platform in China, to conduct questionnaire distribution. Because the subjects in this study were those who had watched pet videos, the questionnaire was distributed and collected by the snowball sampling method from August 10 to 30, 2020. Specifically, first, social relations such as relatives, friends, colleagues, classmates and students who had watched the pet animal videos or livestreams were invited to fill in the questionnaire, and then they were asked to recommend and invite those they know who had watched pet videos and have online pet watching experience to fill in the questionnaire. In addition, to further ensure the validity of the respondents, we included the question “have you ever watched a pet video?” If they answered no, the questionnaire was immediately closed. We invited a total of 547 people to complete this questionnaire, but 108 of them had never watched pet videos online. Finally, we obtained 439 valid questionnaires. It is worth mentioning that we have considered the possible endogeneity of the impacts of online pet watching on watchers’ subject-wellbeing. The respondents’ watching frequency, involvement degree, and attitudes towards online pets are heterogeneous although they all had the experience of watching online pet videos/livestreams. They may only have watched online pets occasionally, or they used to watch online pet videos/livestreams. Moreover, their emotional responses to online pet watching also vary from the passionate to the normal. In general, the respondents do not necessarily have high involvement and positive attitudes towards online pets. In other words, there is no noticeable preexisting preferences among participants that may lead to positive effects on subject-wellbeing. By selecting the heterogenous samples, we have tried our best to minimize the effect of endogeneity.

Besides, we also collected data through semi-structured interviews. We interviewed 12 individual participants who engaged in online pet streaming (see Table 1) to understand people’s true feelings and experiences of watching online pet videos/livestreams, and to offer a supplementary explanation to the results of this study, in order to make our research more substantial and solid. The interviews conducted with the audiences mainly covered (but were not limited to) the following eight aspects: (1) the actual feelings of telepresence in watching pet videos/livestreams, and how the telepresence in online pet watching affect subjective well-being; (2) the actual feelings of social presence in watching pet videos/livestreams, and how the social presence in online pet watching affect subjective well-being; (3) how the telepresence in online pet watching affect flow experience; (4) how the social presence in online pet watching affect flow experience; (5) how the flow experience in online pet watching affect subjective well-being; (6) the actual feeling of pet watching under different levels of loneliness; (7) the actual feeling of pet watching under different levels of perceived pressure; (8) the difference between online pet watching and “offline” pet keeping. Interview data is presented in the discussion in order to comprehensively capture the social processes underlying the quantitative causality. 

### 3.3. Data Analysis

In this paper, SPSS 22.0 (IBM Corporation, Armonk, NY, USA) was used to understand the basic attributes of the survey samples and the reliability and validity of each research variable. The structural equation modelling software AMOS 24.0 (IBM Corporation, Chicago, IL, USA) was used to conduct confirmatory factor analysis, model parameter estimation, model matching and hypothesis testing to verify the relationships among telepresence, social presence, flow experience and subjective well-being.

## 4. Results

### 4.1. Characteristics of the Respondents

Among the 439 valid samples, 112 were male, accounting for 25.5%, and 327 were female, accounting for 74.55%. In terms of age, most of them were young people aged 21–30, accounting for 83.4%. More than 50% of the respondents had a monthly income of less than 3000 yuan. More than 65% of the respondents had the experience of keeping pets, but only 27.6% of them had pets at the time. Most of the respondents (80.6%) said they spent less than 20 minutes watching pets online every day, and more details are shown in Table 2. According to the China Pet Industry White Paper of 2019, the proportion of women keeping pets is higher, and from the perspective of age, the proportion of the post-90s population keeping pets has increased rapidly, including 35.6% of the post-95s population that keeps pets. The urban pet owner population shows a trend of being younger and having higher education and higher income. Our sample characteristics are consistent with the white paper results.

### 4.2. Reliability and Validity of Measurement Scales

As shown in Table 3, good reliability was demonstrated for the five measurement scales. All had a higher than 0.5 average variance extracted (AVE) and greater than 0.7 Cronbach’s α and composite reliability coefficients, which means that the scales used in this paper all have great reliability and validity.

### 4.3. Structural Equation Modelling (SEM) Results

The maximum likelihood estimation method in AMOS 24.0 software (IBM Corporation, Chicago, IL, USA) was used to test the conceptual model. As shown in Table 4, the results of the structural equation model analysis showed that the fitting indexes of the data and model were as follows: χ^2^/df = 2.730, RMSEA = 0.063, GFI = 0.930, CFI = 0.967, IFI = 0.968, RFI = 0.934, TLI = 0.957. All the main indexes are greater than or close to the best standard of 0.9. The fitting indexes of the structural model reached a good level, the overall fitness level is good, and the model has a good degree of fit.

### 4.4. Structural Model

**Figure 3:**
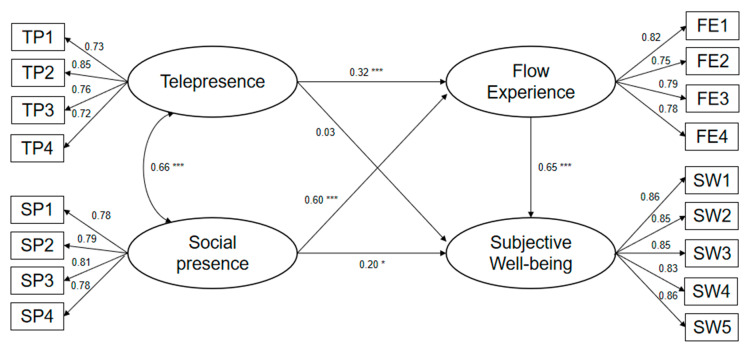
Results of structural model testing. *** Significance at the 0.001 level; * significance at the 0.05 level.

Figure 3 shows a representation of a structural model with a normalization coefficient and a saliency level for each proposed path. The results show that telepresence and social presence both have positive effects on flow experience, and social presence can also directly affect subjective well-being. In other words, online interaction in online pet watching can affect people’s subjective well-being. The flow experience in watching online pet videos/livestreams has a strong influence on subjective well-being, and its standardized path coefficient reaches 0.65. In sum, the presence which is generated by online pet watching has a positive impact on subjective well-being, but it is mainly achieved by influencing flow experience firstly. It shows that flow experience is a very key variable in online pet watching, a new digital leisure activity, and the flow level directly determines whether this activity can bring subjective well-being to people.

### 4.5. The Moderating Effect of Loneliness and Perceived Stress

This study used a stepwise hierarchical regression analysis to test the moderating effect of perceived stress and loneliness. To test the moderating effect of loneliness between social presence and flow experience, we put the control variables (gender, age, education, watch time) in the first step; the independent variable (social presence) in the second step; the adjustment variable (loneliness) in the third step; and the normalized independent variable and the adjustment variable product interaction term in the fourth step. The results showed that loneliness had a positive moderating effect on the relationship between social presence and flow experience in the sample (beta = 0.077, *p* < 0.05), and the results support Hypothesis 6; see Table 5.

To test the moderating effect of perceived stress between telepresence and flow experience, we put the control variables (gender, age, education, watch time) in the first step; the independent variable (telepresence) in the second step; the adjustment variable (perceived stress) in the third step; and the normalized independent variable and the adjustment variable product interaction term in the fourth step. The results showed that perceived stress had a negative moderating effect on the relationship between telepresence and flow experience in the sample (beta = −0.076, *p* < 0.05), and the results support Hypothesis 7; see Table 6.

**Figure 4:**
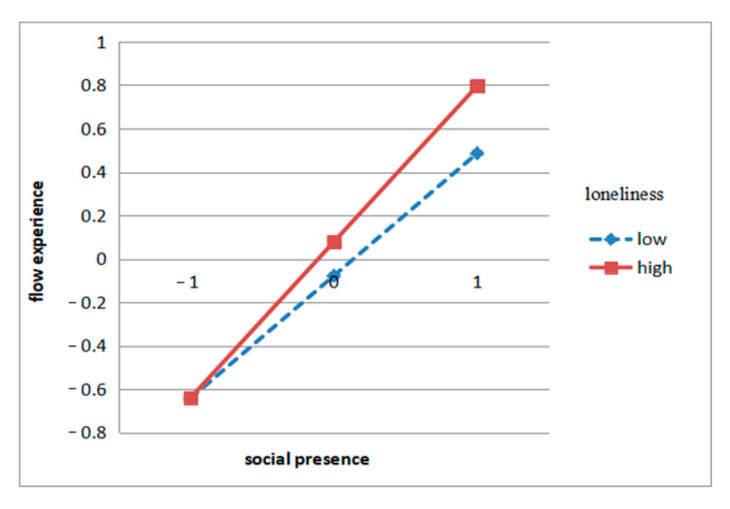
Interaction effects of loneliness and social presence on flow experience.

**Figure 5:**
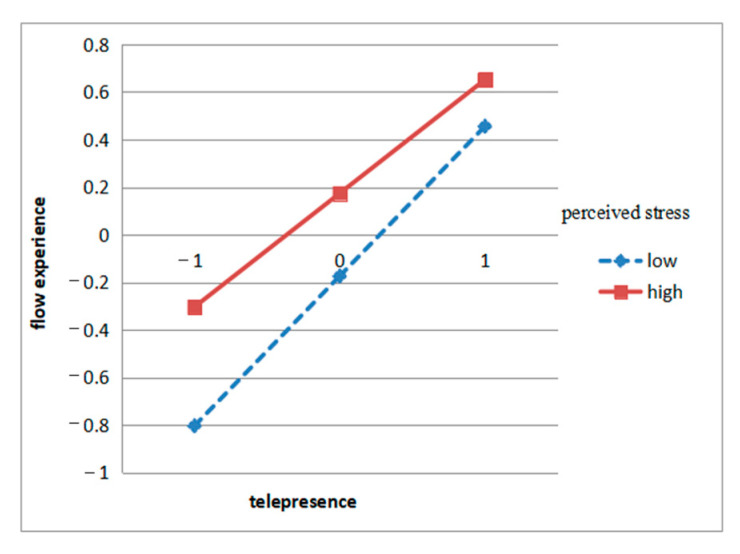
Interaction effects of perceived stress and telepresence on flow experience.

According to the regression equation results, loneliness had a positive moderating effect on the relationship between social presence and flow experience, which means that in the case of high loneliness, social presence may have a stronger influence on flow experience. Additionally, perceived stress had a negative moderating effect on the relationship between telepresence and flow experience, which means that when people perceive a high level of stress, the effect of telepresence on flow experience may be weakened. See Figure 4 and Figure 5.

## 5. Discussion

### 5.1. Discussion of Results

The research hypotheses were tested, and the outcomes are showed in Table 7. The results show that the presence feeling while watching pet videos/livestreams has a positive impact on people’s subjective well-being, and the flow experience plays an important role as a transmitter and a mediator between them.

The hypothesis H1 was rejected, and it means telepresence which is generated during watching pet videos/livestreams has no direct effect on subjective well-being. In other words, just “being there” cannot make people satisfied. Telepresence is defined as the presence experience in the environment through the communication medium [32], which is the degree to which people feel that they exist in the intermediary environment rather than in the direct physical environment. Loomis [64] pointed out that presence is a basic state of consciousness, which is a part of sensation attributed to some remote stimuli, or to some environment. And this kind of “externalization” and “remote attributes” of consciousness still has some distance to affect subjective well-being.

The hypothesis H2 was supported, and the social presence has a positive effect on subjective well-being. Research on the “the media equation” has suggested that media users usually respond to media content as if it happened in real life [65], so we believe that the media exposure to pets could possibly result in similar outcomes. The level of social presence depends on the interaction between the media and the task at hand and is based on the user’s subjective judgment, which generally involves immediate feedback capabilities, personalization, information richness, etc. [66]. Research has found that visual media can bring more social presence to users [67], and intimacy and immediacy are also two important concepts that affect social presence. The interaction in online pet watching is an online social activity, and this kind of social presence makes people feel accompanied. During watching online pet videos/livestreams, we will feel we are doing something together, and our online speech will get timely feedback from other netizens. All these characteristics of online social contact can help to improve the subjective well-being of online pet watchers. Additionally, interviewee No. 3 said that pet videos gives me a very diverse experience and affect her mood. And according to interviewee No. 5 narrative, he had completely immersed in online interaction during watching pet videos/livestreams, and shared happiness and sorrow with other users.

*“I think watching these pet videos gives me a very diverse experience. Some videos make me feel warmth and healing. For example, a Shiba Inu named Xiaohu in TikTok, which grow up with the pet owner’s son, and their relationship was very intimate. Watching the child embrace the dog with a happy smile, and with the sweet sentimental music, you will feel very warm, or even a little moved to tears. Some videos are funny. For example, dogs run around wearing inappropriate clothes and make you laugh. Of course, all of these will affect my mood.”*

*Interviewee No. 3*

*“Of course, I’ll read these messages and bullet screens in watching pet videos/livestreams. My reactions to different contents are also different. If he comments on the content of the video in a very sympathetic way, for example, if the video content is funny and relaxed, then his comments are also very humorous and funny. If the content of the video is serious, such as calling on us to protect stray animals, his comments are also supportive responses. To this kind of comment, I will feel very good, and I will definitely give him some praise, and even then post my feelings to support him. But for another type of comment, I won’t feel happy. For the deliberate provocation, and even speak evil words to pets, I will be very angry, and scold back.”*

*Interviewee No. 5*

The hypotheses H3 and H4 were supported, which indicated that both telepresence and social presence have positive effects on the flow experience. Together, it means that the information richness and sensory stimulation of social media will eliminate a sense of physical distance, giving people the illusion of being there and generating the flow experience. When audiences watch the pet video and livestream, they will have a close-up view of animals and revel in the pets’ cuteness, as if the pet animals are in front of them. In addition, posting comments while watching pet videos/livestreams is actually a kind of quasi-social interaction, which allows viewers to have the actual feeling of communicating with others, which may bring flow experience, thereby making the audience feel happy. Interviewee No. 10 expressed that online pet watching has a healing effect on himself, and when he was watching online pets, he felt relaxed and enjoyed the present moment.

*“I like pets very very much. I enjoy watching videos in which they play with cats and dogs. Although I can’t keep them by myself because of too much work, I can feel very relaxed and decompressed by looking at other people’s lovely cats and dogs and their daily lovely interactions.”*

*Interviewee No. 10*

The hypothesis H5 was accepted, which was associated with flow experience has a positive effect on subjective well-being, and this research also confirms this argument. We considered that watching pet videos/livestreams can evoke a flow experience and even further improve personal subjective well-being. Existing literature believes that experiencing flow frequently is associated with better well-being [68]. Long term participation in flow-related activities is associated with increased functioning and well-being, such as life satisfaction [69,70]. This research is in line with previous research conclusions and provides a powerful empirical case for it.

The hypothesis H6 was supported, indicating that loneliness has a positive moderating effect on the relationship of social presence on flow experience. This means that with a strong sense of loneliness when people watch pet videos and livestreams, the quasi-social interaction has a stronger effect on the flow experience. In other words, people with high levels of loneliness are more likely to have flow experience through online interactions when watching pet videos. In contrast, people with low levels of loneliness, watching pet videos, making online comments, and interacting with other audiences have less impact on their flow experience. Like most young people working hard in the city, Interviewee No. 7 often feels lonely after getting off work at night. She believed online pet video and live streaming accompany her through the night-time.

*“Especially in the evening, after work, all the tired and lonely upwell in my mind. No matter how late, I want to lie in bed and watch these cute animal videos for a while. After watching, I feel less lonely. The feeling of being with pets and netizens makes me relaxed and enjoyment.”*

*Interviewee No. 7*

The hypothesis H7 was supported, which indicated that perceived pressure had a negative moderating effect on the relationship between telepresence and flow experience. This means that when people are under greater perceived pressure, the influence of telepresence on the flow experience will be weakened. This result is consistent with the consensus in cognitive psychology. Limited attention resources have always been the core concept of cognitive psychology and related applied disciplines [71]. This means that psychological resources are limited, so when under greater pressure, people may find it more difficult to immerse themselves in digital leisure, and it is also difficult to obtain the flow experience in watching pet videos and livestreams. Like what Interviewee No.4 said, when the pressure of his life and work had come, the entertainment videos on the Internet also became boring. He just scanned roughly and couldn’t immerse in it, let alone make himself happy.

*“When I’m under a lot of pressure, I feel that I don’t have the time and energy to concentrate. In particular, most of these cute pet videos are loose and slow-paced. When I’m busy with work, I don’t have the patience to watch them, just take a glance, and then slip away.”*

*Interviewee No. 4*

### 5.2. Theoretical Contributions

This article has made two theoretical contributions. First, this research extends the scope of research regarding pets’ impact on human from physical space to cyberspace; that is, online pets (not limited to physical pets) also have a positive effect on people’s mental health. Traditional studies have suggested that interacting with pets will increase human happiness and provide comfort and support [21]. However, existing research has been limited to the physical interactions of humans and pet animals, rarely involving virtual interactions on the Internet, so our study is a supplement to the research on virtual interactions between humans and pets. Second, this study applies the presence theory to explain the sensual experiences of online pet watchers. In this paper, presence is used as a key variable to construct a theoretical model that provides a new theoretical explanation for the phenomenon of online pet watching. We have revealed the psychological mechanism of online presence on people’s subjective well-being in digital leisure. Third, this research clarified the boundary conditions of how presence affects the flow experience. The existing literature on the impact of pets on humans generally takes stress and loneliness as outcome variables or dependent variables. These studies have assumed that the cognitive presence of pets can reduce the stress response [21,72,73] and relieve loneliness in life [74,75,76], and the effect of pet photos has also been documented [77]. However, this study considered perceived stress and loneliness as moderating variables to investigate the relationship between presence and flow experience. We have explained people’s different responses to online pets under different perceived stress levels and varying degrees of loneliness, which is a valuable exploration.

Some researchers believe that pet owners show healthier and better conditions because keeping pets requires good physical condition and great economic conditions [78]. In other words, they think the good physical condition may be a prerequisite for keeping pets, not the results from having pets. Although online pet watching is a highly accessible activity, the research results showed that this digital leisure activity can significantly help improve personal life satisfaction and subjective happiness. Therefore, this article provides further empirical support for the positive impact of pets on human mental health. In addition, studies have proven that hedonistic behaviors have a stronger positive correlation with positive emotions of happiness [79], and this article found that watching short videos and livestreams, i.e., this new digital leisure, was one type of hedonistic behavior that can also result in greater subjective well-being. Mood management theory believes that personal consumption of media eliminates negative emotions or maintains positive emotions [80,81]. Media use can be seen as a form of emotion regulation [82]. People often unconsciously choose different media content based on affinity, interest, excitement, hedonic value, etc. This is why so many netizens frequently browse videos and livestreams about pets and actively participate in digital leisure.

### 5.3. Practical Implications

This study finds that pet videos have a significant positive effect on subjective well-being. This means that moderate pet video viewing can be regarded as a kind of digital leisure, which can help people improve their sense of happiness and life satisfaction. This fully affirms the positive impact of short videos and livestreams and affirms the important role of Internet pet animals in delighting audiences and providing physical and psychological pleasure. It is worth mentioning that people with a higher sense of loneliness may be more likely to obtain more social support through highly immersive online pet watching and online quasi-social interactions to compensate for social deficiencies in real life. This discovery is of great significance to the physical and mental health of contemporary youth living alone.

According to the empirical results of this study, pet video and live streaming can evoke flow experiences and improve personal life satisfaction. This is useful to understanding people who lack of social and economic capital but resort to online leisure activities for happiness. This is particularly the case for urban youth who often feel lonely, pet videos and livestreams may help you dispatch loneliness and possibly find like-minded online friends. However, for those who are already in a very stressful situation and but intend to utilize pet videos and live streaming as a way to escape from reality, it not only reduces their flow experience and pleasure but also likely makes them more anxious. In addition, online pet watchers are more likely to become addicted to hedonic content if they rely too much on this form of pleasure. They also risk of falling into digital addiction or social media addiction that potentially bring deteriorated effects on their health and normal life. As interviewee 12 said: “I became aware that swiping the screen has occupied my life, because it’s easy to take out my mobile phone to watch the videos about cute company animals. I think it’s easy to get into it and forgot the time, but it can’t bring practical benefits to my life and work. I’m very worried about this”.

### 5.4. Limitations and Future Research

This study also has some limitations. Firstly, the samples in this article represent only a part of Internet users, and it is uncertain whether the results can be generalized to all Internet users or all fans of online pet-related media. Secondly, although we have tried to minimise the effects of endogeneity, this study does not explicitly distinguish respondents’ preference for online pets. The research specifically targets at people who have watched online pet videos/livestreams, and we are only able to demonstrate the effects working on those who have watched online pet videos/livestreams. Future research should focus on people’s online pets preference and how it influence their involvement in online pet videos/livestreams. In addition, we find that online pet has a positive effect on people’s subjective well-being, but we cannot completely infer that online pet has a positive effect on all internet users. Future research can provide a comparative research between those who have or have not watch online pet videos/livestreams and clarify their different effects. Thirdly, most of the samples in this study were women. Therefore, how gender influences the dynamics of online pet watching remains unclear. Moreover, this article uses a self-reported questionnaire method and only investigates and verifies the correlation. The next step is to use experiments and other methods for further exploration of key variables. 

This article is only a preliminary description of the psychological process of users’ attraction to these experiences in the cloud. Future research can classify animals and examine the differences in the characteristics and experience of animal followers with different emotional attributes. For example, is the psychological appeal during the viewing process different for users who like warm dogs and cats with high levels of cold attributes? Psychological techniques can also be used to capture the viewer’s physiological indicators through instruments and to explore the causal relationship between specific variables of cloud pets. Besides, as the interviewee 1 said: “the advantage of the online pet is that what owners show you are always the most cuteness and interesting sides of pets, which makes you happy. But the actual pet-keeping is more complex. Keeping a pet is the same as keeping a child, there will be joy and moved, but also irritability and anger. So all the real interactions will deepen your feelings and strengthen your bond. And no matter how good the online pet is, it still belongs to others, and no matter how cute it is, it will be less tangible company and touch”. So future research could try to further compare the effects of physical pets and online pets on people’s subjective well-being and clarify the difference between them.

## 6. Conclusions

During the watching of pet videos and livestreams, telepresence has a positive effect on viewers’ flow experience, and social presence also has a significant positive effect on the flow experience. Then, flow experience has a significant positive effect on people’s subjective well-being, which means that watching pet videos and livestreams may improve one’s subjective well-being and increase people’s satisfaction with their lives. Besides, we found a positive moderating effect of loneliness in the relationship of social presence on flow experience and a negative effect of perceived pressure in the relationship between telepresence and flow experience. Overall, moderately participating in digital leisure activities, and online pet watching in this case, will help improve people’s flow experience and life satisfaction. 

## Figures and Tables

**Table 1:** Interview information on the participants.

Interview	Gender	Age	Occupation	Pet Watching Time
1	male	30	teacher	2 years
2	female	25	nurse	3 years
3	female	26	civil servant	6 months
4	female	20	student	1 year
5	male	23	worker	1.5 year
6	male	32	manager	2 year
7	female	33	accountant	8 months
8	male	27	engineer	1.5 year
9	female	19	student	3 months
10	female	21	student	5 months
11	male	28	student	2 years
12	female	35	civil servant	1 year

**Table 2:** Sample profile (*N* = 439).

Variable	*n*	%	Variable	*n*	%
Gender			Monthly income yuan		
Male	112	25.5	≤3000	224	57.8
Female	327	74.5	3001–5000	61	13.9
Age			5001–10000	84	19.2
Under 20	20	4.6	>10,000	40	9.1
21–30	366	83.4	Had pets before		
31–40	38	8.7	Yes	286	65.1
41–50	11	2.5	No	153	34.9
51–60	4	0.9	Keeping pets now		
Education			Yes	121	27.6
High school or less	29	6.6	No	318	72.4
Associate’s degree	64	14.6	Watch time per day		
Bachelor’s degree	253	57.6	10 min or less	242	55.1
Master’s degree or above	93	21.2	11–20 min	112	25.5
			20–30 min	66	15.1
			30–60 min	16	3.6
			60 min above	3	0.7

**Table 3:** Reliability and convergent validity of the questionnaire.

Constructs and Scale Items	Factor Loading	Cronbach’s Alpha	Composite Reliability	AVE
**Telepresence**		0.851	0.849	0.585
TF1 I forget about my immediate surroundings when I am watching the pet videos/livestreams	0.734			
TP2 Browsing the pet videos/livestreams often makes me forget where I am.	0.845			
TP3 After browsing the pet videos/livestreams, I feel like I come back to the “real world” after a journey.	0.756			
TP4 Watching pet videos/livestreams creates a new world for me, and this world suddenly disappears when I stop using it.	0.717			
**Social Presence**		0.885	0.870	0.625
SP1 There is a sense of human contact during watching pet videos/livestreams.	0.779			
SP2 There is a sense of human warmth during watching pet videos/livestreams.	0.791			
SP3 When watching pet videos/livestreams, the interaction with the other audience is close	0.813			
SP4 When watching pet videos/livestreams, the interaction with the other audience is emotional	0.779			
**Flow Experience**		0.871	0.864	0.614
FE1 While watching pet videos/livestreams, my attention was focused on the activity	0.818			
FE2 While watching pet videos/livestreams, I concentrated fully on the activity and forgot other things	0.746			
FE3 While watching pet videos/livestreamss I feel time flies	0.790			
FE4 While watching pet videos/livestreams I feel excited	0.777			
**Subjective Well-being**		0.925	0.930	0.728
SW1 After watching pet videos/livestreams I felt that I lead a meaningful and fulfilling life	0.861			
SW2 Overall, watching pet videos/livestreams was memorable having enriched my quality of life.	0.854			
SW3 I felt good about my life shortly after watching pet videos/livestreams	0.853			
SW4 Overall, I felt happy upon my return from watching pet videos/livestreams.	0.835			
SW5 My satisfaction with life in general was increased shortly after watching pet videos/livestreams.	0.862			
**Loneliness**		0.886	0.898	0.751
LO1 In general, I feel like I lack companionship.	0.659			
LO2 In general, I feel like I am often left out of social situations.	0.996			
LO3 In general, I feel isolated from others.	0.910			

**Table 4:** Goodness-of-fit indexes.

Model-Fit Index.	Absolute Index					Comparative Index			Parsimony Index		
	CMIN/DF	GFI	RMR	AGFI	RMSEA	IFI	TLI	CFI	PGFI	PNFI	PCFI
Threshold value	<5	>0.90	<0.05	>0.90	<0.08	>0.90	>0.90	>0.90	>0.50	>0.50	>0.50
Structural model	2.730	0.930	0.034	0.897	0.063	0.968	0.957	0.967	0.632	0.726	0.740

Note. CMIN/DF: chi-square/degree of freedom; GFI: goodness-of-fit index; RMR: root mean square residual; AGFI: adjusted goodness-of -fit index; RMSEA: root mean square error of approximation; IFI: incremental fit index; TLI: Tucker-Lewis index; CFI: comparative fit index; PGFI: parsimony goodness-of-fit index; PNFI: parsimony normed fit index; PCFI: parsimony comparative fit index.

**Table 5:** Loneliness, social presence and flow experience structure.

Variables	1	2	3	4
**Control variable**				
gender	0.078	0.062	0.069	0.070
age	0.010	−0.002	0.004	0.003
education	0.104 *	0.113 **	0.124 ***	0.129 ***
watch time	0.311 ***	0.159 ***	0.153 ***	0.154 ***
**Independent variables**				
social presence		0.647 ***	0.623 ***	0.641 ***
**Moderator**				
loneliness			0.097 **	0.078 *
**Interactions**				
social presence*loneliness				0.077 *
				
R^2^	0.114	0.510	0.518	0.524
ΔR^2^	0.114	0.396	0.008	0.005
F	13.993 ***	90.075 ***	77.466 ***	67.667 ***

Dependent variable: flow experience * Significant at *p* < 0.05. ** Significant at *p* < 0.01. *** Significant at *p* < 0.001.

**Table 6:** Perceived stress, telepresence and flow experience structure.

Variables	1	2	3	4
**Control variable**				
gender	0.078	0.066	0.073	0.066
age	0.010	0.036	0.030	0.028
education	0.104 *	0.086 *	0.085 *	0.073 *
watch time	0.311 ***	0.189 ***	0.187 ***	0.187 ***
**Independent variables**				
telepresence		0.606 ***	0.549 ***	0.554 ***
**Moderator**				
perceived stress			0.160 ***	0.174 ***
**Interactions**				
telepresence*perceived stress				−0.076 *
R^2^	0.114	0.466	0.488	0.493
ΔR^2^	0.114	0.351	0.022	0.005
F	13.993 ***	75.478 ***	68.655 ***	59.950 ***

Dependent variable: flow experience * Significant at *p* < 0.05; *** Significant at *p* < 0.001.

**Table 7:** Summary of hypotheses testing results.

Hypotheses				SRW	Outcomes
H1	Telepresence	→	Subjective well-being	0.03	Rejected
H2	Social presence	→	Subjective well-being	0.20 *	Accepted
H3	Telepresence	→	Flow experience	0.32 ***	Accepted
H4	Social presence	→	Flow experience	0.60 ***	Accepted
H5	Flow experience	→	Subjective well-being	0.65 ***	Accepted
H6	Loneliness moderates the relationship between social presence and flow experience			0.077 *	Accepted
H7	Perceived stress moderates the relationship between telepresence and flow experience			−0.076 *	Accepted

Note. *** Significance at the 0.001 level; * significance at the 0.05 level.
